# Implementation failures in the use of two New Zealand laws to control the tobacco industry: 1989–2005

**DOI:** 10.1186/1743-8462-2-32

**Published:** 2005-12-14

**Authors:** George Thomson, Nick Wilson

**Affiliations:** 1 Department of Public Health, Wellington School of Medicine and Health Sciences, University of Otago, Box 7343 Wellington South, New Zealand; 2 Department of Public Health, Wellington School of Medicine and Health Sciences, University of Otago, Box 7343 Wellington South, New Zealand

## Abstract

**Background:**

We reviewed the implementation of New Zealand laws in relation to the activities of the tobacco industry and their allies. Material for two brief case studies was obtained from correspondence with official agencies, official information requests, internet searches (tobacco industry documents and official government sites), and interviews with 12 key informants.

**Results:**

The first case study identified four occasions over a period of 14 years where New Zealand Government agencies appeared to fail to enforce consumer protection law, although apparent breaches by the tobacco industry and their allies had occurred in relation to statements on the relative safety of secondhand smoke. The second case study examined responses to a legal requirement for the tobacco industry to provide information on tobacco additives. There was failure to enforce the law, and a failure of the political process for at least 13 years to clarify and strengthen the law. Relevant factors in both these cases of 'policy slippage' appear to have been financial and opportunity costs of taking legal action, political difficulties and the fragmented nature of government structures.

**Conclusion:**

Considered together, these case studies suggest the need for governments to: (i) make better use of national consumer laws (with proper monitoring and enforcement) in relation to tobacco; and (ii) to strengthen international law and resources around tobacco-related consumer protection. A number of options for achieving these aims are available to governments.

## Introduction

A number of authors argue that specific attention by governments and advocates to the policy implementation process is needed in tobacco control, to avoid the risk of 'policy slippage' [1-3]. Policy slippage is the gap between the intent of policy in legislation or government directives, and the implementation of such policy. This paper investigates the way two national laws relevant to the activities of the tobacco industry were implemented. One law concerns deceptive statements to potential consumers and the public, the second, the disclosure of tobacco additives. The setting is New Zealand, which has some components of a comprehensive tobacco control programme [4,5].

## Background

The New Zealand tobacco control programme has over the last 15 years introduced tobacco promotion controls, smoking bans in nearly all indoor work and public places, a free Quitline cessation service, and access to heavily subsidised nicotine replacement treatment. There have also been active non-government tobacco control groups since the early 1980s [5,6]. Although per capita consumption has declined over the last decade, until 2004 smoking prevalence has stayed at around 25–26% [[7] p.9]. This may be due to high uptake of smoking by youth and the lack of fiscal incentives to support quitting (ie, there has been only two tax rises on cigarettes beyond the rate of inflation since 1991 [8]).

The programme operates in the context of the activity in New Zealand of three major international tobacco companies – British American Tobacco, Philip Morris, and Imperial Tobacco. The activities of such companies have been part of the impetus for the recent formation (and ratification by most countries) of the Framework Convention on Tobacco Control, the first treaty under the World Health Organization's treaty making powers.

## Methods

Brief case studies were used to review the implementation of the two laws. The first case, around the *Fair Trading Act 1986*, involved the collection of material from: correspondence with official agencies, requests to the New Zealand Government (using the Official Information Act process), the opportunistic receipt of an official memorandum sent to a Member of Parliament, a search of the New Zealand Commerce Commission's website for media releases issued from 1994 to September 2002 [9], and a search of the Factiva media website for 'New Zealand major newspapers' for the period September 2001 – August 2002, using the phase 'Commerce Commission' and the word 'court'. The period covered for the case study was from 1989 to 2004.

The second case, concerning the provision for tobacco additives disclosure in the *Smoke-free Environments Act 1990*, involved website searches for both tobacco industry and New Zealand Government correspondence. The tobacco industry document websites tobaccodocuments.org, Legacy Tobacco Documents Library, and British American Tobacco Documents Archive were searched for material on the disclosure of tobacco additives in New Zealand, using the searchwords 'zealand' 'additives' 'ingredients' 'Wellington' and 'Department/Ministry of Health' and then by following names revealed by the search. The period covered was from 1990 to 2004.

For both cases, material from interviews with four government officials and ex-officials, two academics, four politicians and two lawyers was used to assess the validity of the documentary evidence, to provide leads for inquiries, and to provide policy and political context.

## Results

### Context

Two aspects of the context for the case studies were: a lack of priority for tobacco control by the dominant party in government during 1991–1999 (the conservative National Party) [10], and the wish by all governments during the period covered to be seen as 'business friendly' [[11] p.40; [12-14]]. Government officials and ex-officials who were interviewed stated that during the 1990s they were given very clear signals that tobacco was not a priority area for the government. These signals in turn resulted in internal Ministry of Health decisions on the allocation of resources.

Secondhand smoke (SHS) is estimated to cause over 300 deaths per year in New Zealand [15] making it the most important environmental health hazard. Accurate knowledge about the extent of SHS harm is important, as it affects smoking behaviour [16,17]. In addition, the tobacco industry has demonstrated the importance of obscuring public knowledge of SHS harms by their investment in deception about it [18-22]. In 2000, the Minister of Health wrote to the Hospitality Association of New Zealand (HANZ), noting that 'It is extremely important to treat what is said about SHS ... with due caution. ... Continuing statements by the tobacco industry, in particular, that the science is not in on SHS ... have [been] shown to be untrue ...' (King A. *[Letter to Bruce Robertson of the Hospitality Association of New Zealand from the Minister of Health]*. Wellington: 25th October 2000).

Tobacco additives are important because of their role in tobacco product design to increase sales, and because of their potential role in addiction [23-27]. Public *knowledge *about the additives is important to help ensure informed consumers [28-30].

### Case study 1: The tobacco industry and the Fair Trading Act 1986

The *Fair Trading Act *states that:

'No person shall, in trade, engage in conduct that is liable to mislead the public as to the nature, ... characteristics, [or] suitability for a purpose, of goods' [[31] s.10].

The Commerce Commission has stated that its job is to enforce 'legislation that ... prohibits misleading and deceptive conduct by traders' [32]. The Commission has suggested that, on the basis of court decisions, businesses must meet a fairly high standard to comply with the *Fair Trading Act*. The standard of business behaviour expected under the Act includes protecting: 'the gullible, [those] of less than average intelligence or poorly educated.' Generally, the question of *intent *is not relevant, 'rather the issue is whether their actions did or could deceive or mislead'. The Commission has suggested that the Act also applied to 'conduct *likely *to mislead or deceive', (emphasis added) [33].

Over the period 1989–2003, we found four episodes (in 1989, 2000, 2001 and 2002) where the Government had an opportunity to apply this law to the statements of tobacco companies and their allies about SHS. In 1989, the advocacy group ASH NZ wrote to the Commerce Commission about advertisements by the Tobacco Institute of New Zealand, which had stated that 'science has not established that other people's cigarette smoking causes diseases in non-smokers'. The Commission replied that it was 'extremely difficult to accurately gauge the effect of the Institute's advertising campaign and therefore whether the campaign has or is likely to have been misleading and deceptive.' The Commission also noted that 'the cost of any legal action would be considerable' [34].

In 2000, a new Minister of Consumer Affairs asked her officials about the use of the *Fair Trading Act *for legal action against the tobacco industry. She then reported that such 'action may not be possible under this Act' [35]. A request to the Ministry of Consumer Affairs under the Official Information Act for the advice given to the Minister was declined (Manch K. *[Letter from Ministry of Consumer Affairs to George Thomson]*. Wellington: August 23, 2001).

In July 2001, we sent information to the Commerce Commission about the public statements of British American Tobacco (BAT) New Zealand. The material included comments from 1998 and 1999 by BAT officials about the IARC (International Agency for Research on Cancer) SHS study, that:

'The study confirms a view that the industry had long held that while smoke in the air may annoy some non-smokers, passive smoke is not a lung cancer risk' [36].

and more generally that:

'The overwhelming majority of [independent] studies found no overall meaningful increase in risk for those married to a smoker' [37].

These statements misrepresented the IARC findings [38]. Statements to the media in 2001 by HANZ were also given to the Commission, on the basis that the statements could be misleading and deceptive about the dangers of using HANZ members' premises where there was SHS. The statements included:

' A seven year study by the World Health Organisation found no links between passive smoking and health risks' [39] and

'... science has not established a link between passive smoking and cancer' [40].

The statements by BAT and HANZ about SHS were forwarded because they appeared to meet four criteria that could help the Commission make a legal case – a pattern of activity, clear deception, sufficiently recent date, and large potential consequences to public health. The statements appeared to deny or obscure the harm caused by SHS, in a manner that could be described as misleading about tobacco products sold, or the safety of services provided by HANZ members.

The Commission replied that although the material 'appears to be a breach of the *Fair Trading Act*, we will not be investigating it in more depth at this stage' and that the Commission targeted 'issues and trading practices that have the greatest potential detriment to consumers' (Gibson D. *[Letter to George Thomson from the Commerce Commission (FTWN 49513)]*. Wellington: Commerce Commission; August 30, 2001). A spokesperson was reported as saying that the Commission 'had decided to refer the complaint to the Ministry of Health and would take no further action'. This was because 'it is dealt with better under their legislation and they have the staff with the knowledge and expertise' [41].

In reply to the information about statements about SHS that appeared to contravene the *Fair Trading *Act, the Ministry of Health pointed out that the:

'Public Health Directorate lacks the staff and financial resources that would be needed to investigate the examples you presented in detail' but 'in general the Ministry supports the interpretations you present' (ie, that BAT and HANZ appeared to have contravened the Act's sections 9 and 13)

(Matheson D. *Letter to George Thomson from the Deputy Director General, Public Health Directorate (PP70-15-2)*. Wellington: New Zealand Ministry of Health; July 30, 2002).

However, because the Ministry considered that the statements 'were not made in a trading context' and because of resource reasons, the Ministry decided that 'it would not be profitable' to pursue the matter under the *Fair Trading Act *(ibid).

In May 2002, a politician from a minor party outside the government provided information to the Commerce Commission about deceptive practices by the industry. The information included two reported statements from the media during 2001. One, by a BAT official was that 'we have gone through the international evidence on secondhand smoke and there is a pattern of research [indicating] that it is not a serious health issue. Nothing is risk-free in this world. There is a small risk to young people and babies ...' [41]. The Director of the Commission's Fair Trading section wrote of this statement 'reasonable members of the public are unlikely to be misled into believing that this statement suggests that the death of adults is not a serious health risk, or that it denies that adults die from second hand smoke .... The statement represents an opinion and is unlikely to breach the Act' (Battell D. *Memorandum to Fair Trading Committee: Tobacco companies – referral from Sue Kedgley MP*. Wellington: Commerce Commission; September 11, 2002).

The second reported statement sent to the Commission was that the science on SHS showed that 'if there was a risk, and there may be a risk, it's not a large one'. Commenting on this in September 2002, the Director of the Commission's Fair Trading section wrote that it was 'more an expression of opinion than fact. .... The people buying cigarettes are unlikely to be misled by comments on the harm or otherwise of passive smoking' [42].

To put the actions and statements of the Commission into context, we examined the legal actions it *did *initiate under the *Fair Trading Act *during the year to August 2002. In that time the Commission prepared or undertook at least twenty legal actions under the Act about deceptive statements. The issues were all relatively minor in terms of health risks to consumers, and included the claim of a fish and chip shop that it used cholesterol-free oil, eg. [43-46]. The Commission took at least eight other cases to court on competition matters, including two Court of Appeal and one Privy Council case [47-49]. The Commission also decided that it was justified in investing considerable resources in the control of the *competitive *aspects of the tobacco industry, by lengthily contesting a tobacco company merger [50].

Over the period 1994–2004, the most serious breach of the *Fair Trading Act *(in terms of mortality) where the Commission *has *responded, was on children's cots. These were implicated in the deaths of 22 children between 1985 and 1994 [51] (ie, around two deaths per year). In contrast, the total national death toll from SHS in year 2000 alone was estimated at 325 [15].

In September 2005, the BAT New Zealand website continued to cast doubt on the conclusions of the IARC SHS study, and stated:

'... we don't believe that [SHS] has been shown to cause chronic disease, such as lung cancer, cardiovascular disease or chronic obstructive pulmonary disease, in adult non-smokers. ....' [52].

### Case study 2: The non-disclosure of tobacco additives by brand

In August 1990, the New Zealand *Smoke-free Environments *(SFE) *Act *was passed by Parliament. Section 35 of the Act required tobacco companies to submit an annual return showing the weight of all additives used in each tobacco product. In early October 1990 the SFE *Regulations *were passed by an Order in Council, just before an election changed the government. These regulations, amongst other things, set out the way that Section 35 was to be carried out. The regulation stipulated that companies give the weight of every additive for every *product *and then the quantity of '*each brand and each brand variant sold*'. Thus, while 'product' could be considered to be a cigarette brand or brand variant, the regulations could also give the impression that a 'product' was a generic type, such as cigarettes, cigars, or roll-your-own tobacco.

From then to the present, tobacco companies operating in New Zealand have endeavoured to continue to both keep the additives *used in New Zealand *secret from the public, and to not disclose to government the additives *for particular brands*. In late 1990, a Philip Morris official in Australia reported to the USA on some tactics he might use to get the additives disclosure law changed, and concluded 'short of sinking N.Z. if you have any other ideas, could you let me know' [53].

In 1991 the Government decided to insist on the disclosure of additives by 'each brand and brand variants' [54,55]. Negotiations dragged on, and by December 1992, the Department of Health (DoH) was accepting a 'temporary' compromise position where they accepted information only for all brands, rather than for particular brands [56]. This position was valued by the tobacco companies, and Philip Morris wrote to the Tobacco Institute of New Zealand in 1993 to emphasise that they thought the DoH:

'under the current regulations, could impose brand-by-brand disclosure .... The DoH could become irritated with the industry and simply impose a brand-by-brand disclosure' [57].

A 1993 note by Tony Andrade of the tobacco industry law firm of Shook, Hardy & Bacon also stated that the:

'ingredients law in New Zealand would require complete disclosure of individual ingredients and amounts by brand if strictly enforced' [58].

A Rothmans (NZ) official warned a RJ Reynolds official that for the 1994 return:

'there is a real possibility that the Department of Health will insist on a return in the correct form, that is, identifying the additives actually present rather than listing over 2000 possible additives. They may decide ... that we will be prosecuted if we file an incorrect return again. We will dispute this, but we would much prefer that it does not come to this. ... We have argued with them for over three years and their patience together with industry credibility must be close to breaking point' [59].

The government had three options in relation to implementing the SFE regulations: to compromise in some way; to insist on information by each brand (and prosecute if necessary); or to make clearer regulations that put the industry's obligations beyond dispute. There was little will by the political party in power until 1996 to confront the tobacco industry [10] and the compromise position was continued.

In March 1994, the New South Wales Cancer Council in Australia released the list of additives given to the New Zealand Government by the tobacco companies. This list had been obtained by a request by ASH New Zealand, under the New Zealand Official Information Act [60]. This release, along with the ongoing risk of further New Zealand Government action on disclosures, appears to have resulted in at least two international level meetings of the tobacco companies involved [61,62].

After the election in late 1996, the political balance was slightly altered. The new Associate Minister with responsibility for tobacco control, Neil Kirton, was a member of the new minority party (New Zealand First) in the ruling coalition. After the four-year period of the compromise which did not insist on disclosure by brand, in August 1997 the Ministry of Health wrote to the tobacco companies, requiring information on additives for each brand [63]. However, that month Kirton was dismissed as Associate Minister, and successive Ministers responsible for tobacco control did not pursue the matter as a priority.

An official described the problem from the bureaucracy's point of view:

'It's basically an issue of resourcing. ... Any regulation of the tobacco industry is very confrontational. It requires a lot of servicing in terms of the kind of official information that is requested and the kind of expectations around consultation.'

Finally in 2003, the new amendments to the Smoke-free Environments Act required the testing and disclosure of each separate brands and brand variant, 'as the regulations may require'. In September 2005, these regulations were apparently being developed but their contents were unknown.

## Discussion

### Main findings and interpretation

The two cases illustrate the sub-optimal implementation of existing laws that could have aided the control of a product that is a major cause of premature death and health inequalities in New Zealand. Possible reasons for this level of implementation of legislation relevant to tobacco companies include: the financial and opportunity costs of implementation, the political difficulties involved in confronting an aggressive industry, and the fragmented nature of government. We considered that the Ministry of Health's statement that the statements on SHS 'were not made in a trading context' as untenable, as the context for the sale of tobacco include the health claims made for the products.

The two cases illustrate the importance of the costs of implementation when governments are faced with very large transnational companies, known to be tenacious litigators willing to use every aspect of the legal process. They also illustrate the need for substantial investment in an aggressive comprehensive tobacco control programme, to ensure that progress is made in spite of difficulties or hesitancies in the enforcement of relevant laws.

When the *Fair Trading Act *issues arose, the relevant government agencies appeared to first think about their budgets and staff resources. In 1989 and 2001, the costs of legal proceedings and the staff resources needed were factors explicitly mentioned by the Commerce Commission. In contrast, the Commission prosecuted a number of other (often small) businesses for minor matters, and were willing to take tobacco and other large companies to court on *competition *matters. This may indicate a greater focus within the Commission on market regulation compared to the health of consumers.

The Ministry of Health stated explicitly that it lacked 'the staff and financial resources that would be needed to investigate the examples you presented in detail' (Matheson D. *Letter to George Thomson from the Deputy Director General, Public Health Directorate (PP70-15-2)*. Wellington: New Zealand Ministry of Health; July 30, 2002) let alone to prosecute tobacco companies on this issue. With the additives issue, officials knew that any attempt to enforce the law would take scarce staff time and budgets just for consultation and Official Information Act request processing, even before any legal expenses.

The fear of high legal costs is justified by the experience of governments in Canada [64], the USA [65,66], the European Union [67] and elsewhere, where tobacco companies use all procedural and appeal avenues in court actions. The intended effect is partly to discourage future litigation against them.

Internationally, government agencies generally find that effective investment in long-term population health issues can be squeezed out by short-term issues on political agendas [68-70]. While the New Zealand Ministry of Health has a large operational budget, any legal costs in an action such as this would come from the funds of the Public Health Directorate. This Directorate controls less than 3% of the Ministry budget, much of which has been devoted to politically sensitive issues in recent years (Matheson D. *Letter to George Thomson from the Deputy Director General, Public Health Directorate*. Wellington: New Zealand Ministry of Health; 8 July, 2002). In comparison with resources for immunisation and cancer screening, the use of health resources for legal expenses was politically difficult.

The fragmented focus of government also contributed to unwillingness to take advantage of the legislative opportunities. While the Commerce Commission was willing to take tobacco companies to court on monopoly issues, it considered that aspects of the *Fair Trading Act *with health implications were 'dealt with better' by the Ministry of Health [41]. The statement of the Commission staff, that they focused on 'issues and trading practices that have the greatest potential detriment to consumers', may indicate that that they had not realised (or chose to ignore) the health risks from secondhand smoke, and the negative effects on health of continued statements that downplayed those risks.

The Ministry, while it had some focus on *smokers*, was less able to focus on the tobacco industry as a major upstream threat to public health. In New Zealand and elsewhere, there has generally been pressure to invest the health sector budget in the treatment of disease, and action on individual risk behaviours [71], rather than invest in 'non-health' avenues such as legal action against tobacco companies.

The cases indicate that for both deceptive statements about SHS and for the non-disclosure of additives in New Zealand, tobacco companies have been able to assume that the risk of legal action by government was low. Much of the usefulness of law is diminished if those businesses that governments are trying to control are not convinced that government legal action will occur or will be effective [72-74]. The New Zealand Government failed to convince tobacco industry investors and managers that government would consistently use the law to seriously challenge industry activities that affected public health in a substantial way.

The investment in legal resources for tobacco control can be contrasted with the actions of the New Zealand Government in another arena. During the first years of its life from 1993, the government agency Pharmac consistently and successfully undertook multi-million dollar legal actions against the pharmaceutical industry. Some of the credit for the success could be ascribed to the clear focus of the agency in improving the efficiency of pharmaceutical purchasing on behalf of the State, and to its relatively independent decision making structure [75,76].

However, the impetus for the structure and policies of Pharmac was the rapidly increasing cost of pharmaceuticals to the publicly funded health system. By contrast, the net adverse consequences from the dominance by transnational tobacco companies of the local market were seen by government to generally impact on smokers, rather than on government. The advice from Treasury (disputed by some health advocates) has been that the tax revenue from tobacco sales heavily outweighed the direct costs to government from tobacco use. Therefore, governments may have been under the impression that there was little cost incentive to take a 'Pharmac' approach to the tobacco industry.

The situation in New Zealand on misleading statements by the tobacco industry and its allies on SHS, despite the explicit statute law available to address the problem, has been in contrast to the Australian experience. There, a consumer organisation (largely funded by government) in the early 1990s took the local Tobacco Institute to court on the issue of false information about SHS issues. The court required that the industry could not describe SHS as not being shown to be unsafe [77]. The comparative level of action appears to argue for government agencies that are sufficiently funded, and are mandated to act for consumers (including smokers).

In 2005, the Australian Competition and Consumer Commission 'obtained court enforceable undertakings from Imperial Tobacco Australia Limited' to remove misleading descriptors from its products [78]. The much greater practice in Australia compared to New Zealand, of making the tobacco industry accountable in court for their statements and actions, eg, [79-82], may be part of the explanation for the much greater decline in smoking prevalence there [83]. This could be due to the effects of publicity on smoking rates [84]. We have presented the case elsewhere that there may also be a relationship between a government focus on the tobacco industry, and more effective tobacco control [85].

The issue of additives disclosure illustrates some of the consequences of the contrasting national and international perspectives of governments and tobacco companies. The New Zealand Government saw the disclosure as a relatively minor matter, not requiring a high priority. In contrast, the companies are able and willing to focus international resources for long periods in New Zealand [86] and in other countries such as Australia [24], Thailand [87] and the Netherlands [88].

### Limitations of these case studies

These case studies were not exhaustive, but rather intended to illuminate aspects of implementation of tobacco control policy. Interviews with other key informants, and access to further documentation could potentially have provided more in-depth evidence and context. Nevertheless, we consider it likely that the evidence gathered around these case studies covers the major dimensions of the issues involved.

### Policy implications

To more adequately address the type of problems identified in this article, and to reduce the risk of policy slippage, the following options could be considered by many governments:

1) Increasing central government capacity to work together across national boundaries to take effective and coordinated action in relation to tobacco companies [89]. This includes continuing the development of the Framework Convention on Tobacco Control, so as to strengthen international consumer protection law against tobacco-related marketing and misleading claims.

2) Provision of support for an international consumer protection law resource centre in a United Nations organisation (eg, one for health related consumer protection in the World Health Organization).

3) Ensuring explicit responsibility for the implementation of national consumer laws by particular government agencies, and provision of funding to ensure that the laws are adequately monitored and enforced.

4) Explicitly acknowledging the role of the tobacco industry as a factor in tobacco-related harm, and acting to minimise their influence.

Options 2 and 3 are likely to be particularly cost-effective, as they can also assist in dealing with other major and extremely costly threats to public health (eg, the control of obesogenic foods and alcohol) [90,91]. Non-governmental organisations can advocate for all these responses, and use the media to highlight deficiencies in existing consumer protection laws.

The cases suggest immediate actions. In New Zealand, court action is needed to clearly establish the relevance of the *Fair Trading Act *to misleading statements that affect the perceptions of at least some of the public about SHS. The writing of the new regulations, on the disclosure of tobacco product constituents and design, needs to allow for considerable powers by government to address any attempts by the tobacco industry to find loopholes in legislation designed to protect the public interest.

## Conclusion

Existing New Zealand laws on misleading statements about secondhand smoke, and about the provision to government on tobacco additives, appear to have been insufficiently enforced. The health impact of secondhand smoke, and the importance of information for consumers about dangerous products suggest that such enforcement is necessary.

## Competing interests

Both authors have undertaken contract work for tobacco control related non-government agencies and NW has undertaken contract work in tobacco control for the New Zealand Ministry of Health.

## Authors' contributions

GT gathered the great majority of the data and conceived the article. Both authors designed the study, analysed the data and wrote and approved the article.

## References

[B1] Bialous S, Glantz S (1999). Arizona's tobacco control initiative illustrates the need for continuing oversight by tobacco control advocates. Tobacco Control.

[B2] Balbach ED, Traynor MP, Glantz SA (2000). The implementation of California's tobacco tax initiative: the critical role of outsider strategies in protecting Proposition 99. J Health Polit Policy Law.

[B3] Jacobson P, Wasserman J (1999). The implementation and enforcement of tobacco control laws: policy implications for activists and the industry. J Health Polit Policy Law.

[B4] Laugesen M, Swinburn B (2000). New Zealand's tobacco control programme 1985–1998. Tob Control.

[B5] Thomson G, Wilson N, Davis P, Ashton T (2001). Tobacco control policy. Health and public policy in New Zealand.

[B6] Studlar DT (2005). The Political Dynamics of Tobacco Control in Australia and New Zealand: Explaining Policy Problems, Instruments, and Patterns of Adoption. Australian Journal of Political Science.

[B7] Ministry of Health (2003). Tobacco facts 2003: Public Health Intelligence Occasional Report No20.

[B8] Wilson N, Thomson G (2005). Tobacco tax as a health protecting policy: a brief review of the New Zealand evidence. N Z Med J.

[B9] Commerce Commission Media Releases.

[B10] Thomson G, Wilson N (2000). Lost in the smoke: tobacco control in New Zealand during the 1990s. NZ Med J.

[B11] Menz G A Model Strategy for Small States to Cope and Survive in a Globalised World Economy? An Analysis of the "New Zealand Way". European Consortium for Political Research (ECPR): Workshop 26: National Models and Transnational Structures – Globalisation and Public Policy: 26 – 31 March 1999.

[B12] McCully M Forest Owners Association Speech at International Forest Industry Safety Conference.

[B13] Shipley J Address To National Party Conference.

[B14] Asia Pacific Bulletin New Zealand's Election Keeps the Door Open to Business.

[B15] Woodward A, Laugesen M (2001). How many deaths are caused by second hand smoke?. Tobacco Control.

[B16] Blackburn C, Spencer N, Bonas S (2003). Effect of strategies to reduce exposure of infants to environmental tobacco smoke in the home: cross sectional survey. Br Med J.

[B17] Gilpin E, White M, Farkas A (1999). Home smoking restrictions: which smokers have them and how they are associated with smoking behavior. Nicotine Tob Res.

[B18] Diethelm PA, Rielle J-C, McKee M (2005). The whole truth and nothing but the truth? The research that Philip Morris did not want you to see. Lancet.

[B19] Trotter L, Chapman S (2003). "Conclusions about exposure to ETS and health that will be unhelpful to us": how the tobacco industry attempted to delay and discredit the 1997 Australian National Health and Medical Research Council report on passive smoking. Tob Control.

[B20] Samet J, Burke T (2001). Turning science into junk: the tobacco industry and passive smoking. Am J Public Health.

[B21] Barnes D, Bero L (1998). Why review articles on the health effects of passive smoking reach different conclusions. JAMA.

[B22] Barnes D, Hanauer P, Slade J (1995). Environmental tobaccosmoke. The Brown and Williamson documents. JAMA.

[B23] Tuffs A (2005). German scientists demand a ban on tobacco additives. Br Med J.

[B24] Chapman S (2003). "Keep a low profile": pesticide residue, additives, and freon use in Australian tobacco manufacturing. Tob Control.

[B25] Wayne GF, Connolly GN (2002). How cigarette design can affect youth initiation into smoking: Camel cigarettes 1983–93. Tob Control.

[B26] Connolly G, Wayne G, Lymperis D (2000). How cigarette additives are used to mask environmental tobacco smoke. Tobacco Control.

[B27] Vagg R, Chapman S (2005). Nicotine analogues: a review of tobacco industry research interests. Addiction.

[B28] Bates C, McNeill A, Jarvis M (1999). The future of tobaccoproduct regulation and labelling in Europe: implications for the forthcoming European Union directive. Tobacco Control.

[B29] Chapman S, Liberman J (2005). Ensuring smokers are adequately informed: reflections on consumer rights, manufacturer responsibilities, and policy implications. Tob Control.

[B30] May H, Wigand J (2005). The right to choose: Why governments should compel the tobacco industry to disclose their ingredients. Essays in Philosophy.

[B31] Fair Trading Act (1986). New Zealand Government.

[B32] Commerce Commission (2005). What we do.

[B33] Commerce Commission The Fair Trading Act.

[B34] ASH NZ (1989). Commerce Commission back down. Ashcan.

[B35] Bunkle P Address to ASH annual meeting.

[B36] Graham C Now no reason for smoke bans in public places. Tobacco Times.

[B37] Graham C (1998). Double standards cloud the 'passive smoking' argument. Tobacco Times.

[B38] Ong E, Glantz S (2000). Tobacco industry efforts subverting International Agency for Research on Cancer's second-hand smoke study. Lancet.

[B39] Evening Post Bar-goes the target in smoking studies. Evening Post.

[B40] Paltridge A Passive smoking victims may sue. Evening Post.

[B41] Paltridge A Smoking claims a health issue, commission says. Evening Post.

[B42] Battell D Memorandum to Fair Trading Committee: Tobacco companies – referral from Sue Kedgley MP.

[B43] Commerce Commission Commerce Commission considers prosecution (media release 2002/2).

[B44] Commerce Commission Plumbing World fined $10,000 for misleading advertising (Media Release 2002/26).

[B45] Commerce Commission Auckland business fined $5,000 for Asian-made KIWI MADE product (Media Release 2002/16).

[B46] New Zealand Press Association Fish and chip man pleads ignorance. Dominion.

[B47] Dominion Commission loses appeal to Privy Council. Dominion.

[B48] National Business Review Court of Appeal begins hearing claim by Commerce Commission. National Business Review.

[B49] Richardson P, Gault J, Blanchard J Carter Holt Harvey Products Group Ltd – vs – The Commerce Commission.

[B50] Chapple I Tobacco case needs work: High Court. New Zealand Herald.

[B51] Commerce Commission Commerce Commission will visit stores to check cots (Media release 2001/47).

[B52] British American Tobacco New Zealand Environmental tobacco smoke.

[B53] Francis P New Zealand [Letter to Mary Pottorff, Philip Morris International].

[B54] Upton S [Letter from the Minister of Health to Michael Thompson of the Tobacco Institute of New Zealand].

[B55] Upton S [Letter from the Minister of Health to MJ Thompson of the Tobacco Institute of New Zealand].

[B56] Talbot P [Letter to Trevor Smith, Managing Director of Rothmans New Zealand].

[B57] McGrath M New Zealand additives returns [Letter to Michael Thompson of the Tobacco Institute of New Zealand].

[B58] Andrade A Update on active jurisdictions concerning ingredients issues [Memo to Matthew Winokur and Anne Oknoniewski].

[B59] Lorrigan P [Fax to D Cooper Rees of RJ Reynolds Tobacco Company].

[B60] Associated Press Cigarette additives.

[B61] Lorrigan P Minutes of New Zealand Additives meeting held at Denham Place 11 July 1994.

[B62] Lorrigan P Ingredients meeting [letter to Dr JD Heck of Lorillard Tobacco Company].

[B63] Poutasi K Smoke-free Environments Act 1990 – Section 35: Tobacco returns [letter to Samantha Kraftt of Chapman Trip Sheffield Young].

[B64] Spurgeon D (2005). Canadian antismoking law is watered down. BMJ.

[B65] Civil Division U.S Department of Justice. Litigation Against Tobacco Companies.

[B66] Nixon M, Mahmoud L, Glantz S (2004). Tobacco industry litigation to deter local public health ordinances: the industry usually loses in court. Tob Control.

[B67] Watson R (2002). European Court adviser rules against British American Tobacco and Imperial. Bmj.

[B68] Newacheck PW, Benjamin AE (2004). Intergenerational equity and public spending: The United States should embrace a new doctrine of fairness to ensure that vulnerable populations are not forced to compete for resources. Health Aff (Millwood).

[B69] Gloyd S, Suarez Torres J, Mercer MA (2003). Immunization campaigns and political agendas: retrospective from Ecuador and El Salvador. Int J Health Serv.

[B70] Jan S (2003). A perspective on the analysis of credible commitment and myopia in health sector decision making. Health Policy.

[B71] Woodward A (2004). Looking upstream: A user's guide to disease prevention. New Zealand Family Practice.

[B72] Anderson FR (1993). Environmental liability dangers. Corporate Board.

[B73] Gruner RS, Brown LM (1996). Organizational justice: Recognizing and rewarding the good citizen corporation. Journal of Corporation Law.

[B74] Kaltenheuser S (1998). Schmiergeld. Across the Board.

[B75] PHARMAC (1999). Briefing paper for the Minister of Health.

[B76] Braae R, McNee W, Moore D (1999). Managing pharmaceutical expenditure while increasing access. The pharmaceutical management agency (PHARMAC) experience. Pharmacoeconomics.

[B77] Chapman S, Woodward S (1993). Australian court decision on passive smoking upheld on appeal. Br Med J.

[B78] Australian Competition and Consumer Commission ACCC resolves 'light' and 'mild' cigarette issue with BAT and Philip Morris.

[B79] Liberman J, Loff B (2002). Australian court rules against tobacco company in lung-cancer case. Lancet.

[B80] Liberman J (2002). The shredding of BAT's defence: McCabe v British American Tobacco Australia. Tob Control.

[B81] Stewart BW, Semmler PC (2002). Sharp v Port Kembla RSL Club: establishing causation of laryngeal cancer by environmental tobacco smoke. Med J Aust.

[B82] Woodward SD, Winstanley MH (1990). Lung cancer and passive smoking at work: the Carroll case. Med J Aust.

[B83] Durkin S, Germain D, Letcher T (2005). Smoking prevalence and consumption in Victoria: key findings from the 1998–2003 population surveys.

[B84] Laugesen M, Meads C (1991). Advertising, price, income and publicity effects on weekly cigarette sales in New Zealand supermarkets. British Journal of Addiction.

[B85] Thomson G, Wilson N (2005). Directly eroding tobacco industry power as a tobacco control strategy. NZ Med J.

[B86] Additives Reporting – New Zealand [Internal industry memo to tobacco companies operating in New Zealand] October 29, 1997.

[B87] MacKenzie R, Collin J, Sriwongcharoen K (2004). "If we canjust stall' new unfriendly legislations, the scoreboard is already in our favour": transnational tobacco companies and ingredients disclosure in Thailand. Tob Control.

[B88] Associated Press Philip Morris Sues Dutch Government August 28, 2003.

[B89] Wipfli H, Stillman F, Tamplin S (2004). Achieving the Framework Convention on Tobacco Control's potential by investing in national capacity. Tob Control.

[B90] Swinburn BA (2003). The obesity epidemic in Australia: can public health interventions work?. Asia Pac J Clin Nutr.

[B91] Toomey TL, Wagenaar AC (1999). Policy options for prevention: the case of alcohol. J Public Health Policy.

